# Discovering HDAC class II selective inhibitors by multidisciplinary approach

**DOI:** 10.1186/1758-2946-3-S1-P2

**Published:** 2011-04-19

**Authors:** S Lorenzi, A Beccari, D Carettoni, M Rubino, V Nardese

**Affiliations:** 1 DrugDiscovery Dept., Dompé spa, L’Aquila,I-67100, Italy; 2 AXXAM spa, Milano, I-20132, Italy

## 

A number of cellular processes, as cell proliferation, apoptosis and cytoskeleton assembly [1], are regulated by enzymes belonging to the family of histone deacetylases (HDACs). Extended or local changes in chromatin structure are driven by an interplay between histone acetyltransferases (HATs) and HDACs, and both of enzymes are involved in post-translational modifications of histones involving the control of the genes expression [2]. One of the most studied post-modification is the acetylation of the lysine residues in the N-terms of the histone cores mediated by the HDACs enzymes, which contains four distinct subgroups: CLASS I (HDACs 1, 2, 3 and 8), CLASS II (HDACs 4, 5, 6 and 7), CLASS III (SIRTUINS) and CLASS IV (HDAC11).

The enzyme under study was the HDAC7 and due to the absence of specific inhibitors of this enzyme we adopt both ligand-based and structure-based approaches to filter from a 7 million commercial compounds database, an ensemble of molecules to test versus HDAC7.

The starting point of our approach was to collect all Metal-Binding heads (MTH) and all kind of Linker between the MTH and the Capping Groups of all available compounds binding all HDACs, and generate all possible combinations among all three moieties. All compounds of this virtual database have been submitted to a conformational search in which a maximum of 255 conformers have been stored per molecules. This last 3D virtual database has been filtered by a pharmacophore built on the tridimensional structure of the HDAC7 in complex with TSA (PDB ID: 3C10).

The molecules able to satisfy the pharmacophore have been submitted to a docking phase to filter molecules with a high propensity to interact with HDAC7 enzyme. A panel of 960 compounds have been purchased and tested on HDAC7 and the most interesting have been characterized against other HDACs.
